# Beyond Juul: The New Face of Underage Nicotine Addiction - A Survey of College Students

**DOI:** 10.1007/s43441-024-00735-1

**Published:** 2024-12-28

**Authors:** Griffin Riggs, Terry David Church

**Affiliations:** 1 University of Southern California, Los Angeles, USA; 2 Department of Regulatory and Quality Sciences, USC Alfred E. Mann School of Pharmacy and Pharmaceutical Sciences, University of Southern California, Los Angeles, USA

**Keywords:** E-Cigarette, ENDS, Vape, Nicotine, Addiction

## Abstract

**Background:**

Youth nicotine addiction is a major public health concern in the United States. Disposable Electronic Nicotine Delivery Systems (ENDS), or disposable vapes, are commonly sought out by youth despite not having received premarket authorization from the FDA. The objective of this study was to identify factors contributing to underage consumption of disposable ENDS.

**Methods:**

An anonymous survey was deployed to college students to understand young adults’ perceptions and patterns of use of disposable ENDS.

**Results:**

Disposable ENDS are very popular among youth. The results of this study revealed the popular brands, flavors, modes of access, and attractive aspects of disposable ENDS. Survey results combined with information from the literature reveal that disposable ENDS gained popularity in the years following the decline in the popularity of pod-based ENDS, such as JUUL, following strict regulatory action from the FDA.

**Conclusion:**

To ultimately address underage nicotine addiction, the FDA must hold disposable ENDS to the same regulatory standards as other tobacco products and produce regulations specifically targeted at disposable ENDS. The results of this study emphasize the importance of making effective regulatory reform and functional educational resources to prevent young people from initiating the use of disposable ENDS.

## Introduction

Youth nicotine addiction continues to be a major public health crisis affecting young people in the United States. The sale of tobacco products, including Electronic Nicotine Delivery Systems (ENDS), to anyone under the age of 21 is prohibited by the legislation known as “Tobacco 21” passed in December 2019 [1]. ENDS is the term used by the U.S. Food and Drug Administration (FDA) to classify all vapes and electronic cigarettes. These products create an aerosol which the user inhales by heating an “e-liquid” which typically contains nicotine, flavoring, propylene glycol, vegetable glycerin, and other ingredients [2]. Disposable ENDS are all-in-one e-cigarettes containing a battery, heating element, and nicotine e-liquid that are intended to be disposed of after the battery dies. Many disposable ENDS closely resemble popular pod-based ENDS, such as JUUL^®^, but only require a single purchase and no setup to begin use.

Many popular ENDS sold in the U.S. contain high concentrations of nicotine made possible by the use of nicotine salt liquids rather than freebase nicotine liquids used in earlier generations of ENDS [3]. Data shows high ENDS usage by youth with ENDS being the most common tobacco product used by middle and high schoolers since 2014 [4]. According to the FDA and Centers for Disease Control and Prevention (CDC) 2023 annual National Youth Tobacco Survey (NYTS), 7.7% of middle school and high school students polled had used ENDS in the past 30 days [4].

Since FDA’s regulatory crackdowns on pod-based ENDS, such as JUUL, disposable ENDS exploded onto the market, under the regulatory radar, and appealed to the same underage consumers JUUL had previously acquired. In 2019, FDA action led to JUUL removing all flavored pods from the U.S. market, leaving an opening for similar ENDS products sold in fruity, minty, or candy-like flavors, such as the popular disposable ENDS brand Puff Bar™ [3]. This trend is evidenced by the 2023 NYTS results which revealed that 60.7% of youth used disposable ENDS as their usual ENDS device type whereas only 16.1% of these respondents reported “Prefilled or refillable pods or cartridges” as their usual device type [4]. Despite their popularity, many disposable ENDS are not compliant with FDA regulations which poses a risk to users.

To legally market a tobacco product in the U.S., a company must receive a written marketing order from the FDA through one of the following types of submissions: Premarket Tobacco Product Applications (PMTA), Substantial Equivalence (SE) Reports, or Exemption from Substantial Equivalence Requests (EX REQ) [5]. The FDA evaluates the products’ compliance with FDA regulations as well as a public health standard intended to reduce the toll tobacco use takes on public health. Concerningly, very few disposable ENDS sold in the U.S. have received premarket authorization from the FDA. In fact, according to statements released by the FDA, many disposable ENDS would likely not receive market authorization from the FDA even if they filed a PMTA [6]. Many disposable ENDS brands without FDA premarket authorization have not been the target of regulatory action, enabling them to stay on the market. Only some companies, such as Puff Bar, have faced such action in the form of Warning Letters, which have successfully led to the removal of their disposable ENDS from the market. In January 2020, the FDA issued a policy that prioritized enforcement against cartridge-based ENDS sold in flavors other than menthol and tobacco, ENDS products for which the manufacturers fail to take adequate measures to prevent minors’ access, or any ENDS product targeted to minors or likely to promote use by minors [6].

It is crucial to understand young people’s perceptions and usage patterns of disposable ENDS. Several features of disposable ENDS make them attractive to youth users. For example, disposable ENDS continue to be sold in fruity and candy-like flavors which have been found to increase youth interest in beginning to vape, reducing the perception of harm, and leading youth users to take more “puffs”, thus increasing their nicotine intake [7]. Additionally, it is hypothesized that, due to the novelty of disposable ENDS, convenience of use and price may be other driving factors of youth disposable ENDS use. Disposable ENDS offer a simple and commitment-free way to begin vaping by only requiring a single device while pod-based ENDS, such as JUUL require a battery, charger, and e-liquid filled pods that can cost more and require multiple purchases.

The FDA has been unable to properly regulate the rapidly growing industry due to the sheer number of unapproved products on the market. Furthermore, NYTS data cannot deduce aspects such as trends of use, attractive factors of the devices, and popularity of all disposable ENDS brands. The objective of this study is to identify factors promoting underage consumption of disposable ENDS by understanding young adults’ perceptions and patterns of use through surveying college students.

## Materials and Methods

After identifying current gaps in survey data on underage ENDS use and possible risk factors for disposable ENDS abuse by persons under the age of 21, an anonymous survey was developed to gain insight into underage disposable ENDS use. Survey questions were modeled after the NYTS with the addition of questions pertaining to the current gaps in disposable ENDS data. The survey was developed using Qualtrics™ XM, an online survey software, which produced a link that could be distributed to college students. Participants of the survey provided data to determine: (a) the percent of respondents who use disposable ENDS; (b) age of initiation to disposable ENDS and ENDS in general; (c) type of ENDS products used including specific disposable ENDS brands; (d) ENDS flavors used and attractive aspects of disposable ENDS; (e) frequency of use; and (f) methods/price of obtaining.

The survey was approved by the University of Southern California (USC) Institutional Review Board (Study ID: UP-22-00023) and deployed to university students via social media, university email chains, and flyers with QR codes posted around the USC campus. This survey utilized a convenience sample consisting mainly of USC students due to the fact that this was an unfunded study as a part of a senior thesis. College students from other universities were allowed to participate, though large samples from other universities were not feasible due to limitations in funding and resources. The survey opened on March 6, 2022 and closed on February 17, 2023.

Survey data was analyzed using Qualtrics XM software analysis tools to identify trends in responses. Data was initially stratified using two filters to show results for respondents who answered “yes” to being a student and completed the survey in its entirety. Another key filter used in data analysis was for underage respondents, age 18–20. It was hypothesized that respondents in this age group would exhibit similar behavioral patterns regarding ENDS consumption as users under 18 because they likely have similar social and lifestyle influences and neither can legally purchase ENDS. Ultimately, the survey data was grouped into the following categories: underage and used ENDS; underage and used disposable ENDS; and underage and indicated disposable ENDS as their usual device.

## Results

Survey responders were not required to answer every question, which accounts for any discrepancies in the total number of responders for each question.

### Survey Demographics

A total of 231 completed survey responses were collected between March 6, 2022 and February 17, 2023. 222 of these 231 (96.10%) completed survey responses were college students, and therefore relevant for data analysis. 36.94% (82/222) of respondents were male, 62.61% (139/222) were female, and 0.45% (1/222) selected “Other” and indicated that they were gender fluid. 14.86% (33/222) of respondents identified as LatinX and 0.45% (1/222) preferred not to say. Racial breakdown, age range, and class level in university of student respondents are shown in Table 1.

**Table 1:** Distribution of respondent demographics (*N* = 222)

Race	Percent of Responses	Number of Responses
American Indian or Alaska Native	0.00	0
Asian	17.57	39
Black or African American	6.31	14
Native Hawaiian or Pacific Islander	1.35	3
White or Caucasian	59.91	133
More than 1 race	8.11	18
Other	4.05	9
Prefer not to say	2.70	6

Age Range	Percent of Responses	Number of Responses
18–20	82.43	183
21–25	17.57	39

Class Level	Percent of Responses	Number of Responses
First-year student	36.04	80
Sophomore	36.49	81
Junior	18.47	41
Senior	6.76	15
Graduate Student	2.25	5

### Underage ENDS Use

**Fig. 1:**
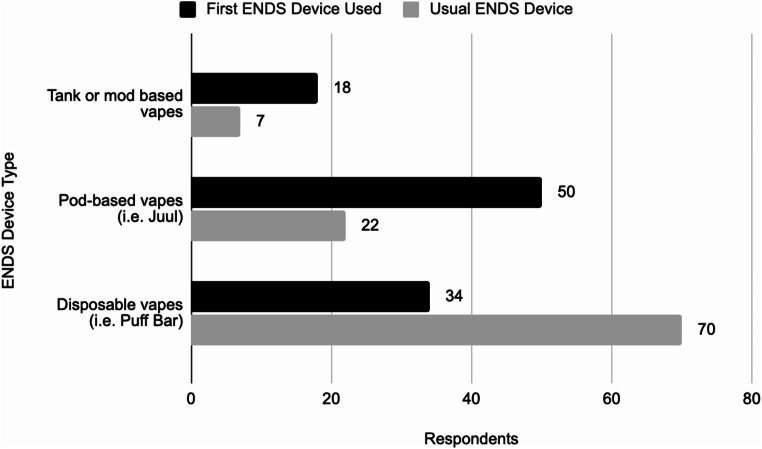
First ENDS device (*N* = 102) and usual ENDS device (*N* = 99) used by underage users

Individuals between 18 and 20 years of age who indicated they had used ENDS at least once in their lifetime represented 55.74% (102/183) of respondents. Figure 1 shows the distribution of the first ENDS device used by all underage respondents who indicated that they had used ENDS at least once. The usual ENDS devices reported by these same underage ENDS using respondents are also shown in Fig. 1. Pod-based ENDS were the most common ENDS device that underage users started with. The age at which participants first used ENDS, regardless of participant age at time of survey completion, is shown in Table 2. This information was important to query from all participants who indicated that they had used ENDS because it revealed that 99.19% (123/124) of respondents used ENDS for the first time while underage.

### Underage Disposable ENDS Use

Underage respondents who had used ENDS at least once in their lives represented 71.57% (73/102) of respondents. The age respondents first used disposable ENDS, regardless of age at survey completion date, is featured in Table 2. Interestingly, just as with ENDS in general, the vast majority, 98.92% (92/93), of survey participants who had used disposable ENDS used them for the first time while under the legal age to use tobacco products in the US. The results of this question allude to the rapidly growing popularity of disposable ENDS around 2018 and 2019 when the majority of respondents indicated they used disposable ENDS for the first time (Table 2). Frequency of ENDS use by both current and past underage disposable ENDS users is shown in Table 3. The 2 respondents who selected “Other” specified: “Only tried it once”; and “Probably 1–2 times per month”.

**Table 2:** Age and year of initiation to ENDS and disposable ENDS

Age of Initiation to ENDS (*N* = 124)	Percent of Responses	Number of Responses
Under 13	0.00	0
13–14	16.94	21
15–16	43.55	54
17–18	27.42	34
19–20	11.29	14
21+	0.81	1

Age of Initiation to Disposable ENDS (*N* = 93)	Percent of Responses	Number of Responses
Under 13	0.00	0
13–14	5.38	5
15–16	26.88	25
17–18	48.39	45
19–20	18.28	17
21+	1.08	1

Year of Initiation to Disposable ENDS (*N* = 93)	Percent of Responses	Number of Responses
2016	4.30	4
2017	10.75	10
2018	22.58	21
2019	30.11	28
2020	13.98	13
2021 or later	18.28	17

**Table 3:** Frequency of ENDS use by underage disposable ENDS users (*N* = 61)

Frequency of Use	Percent of Responses	Number of Responses
10 + times per day	44.26	27
5–10 times per day	14.75	9
2–5 times per day	18.03	11
Once a day	0.00	0
A few times a week	4.92	3
Once a week	3.28	2
Only socially with friends (Please specify about how many times per week)	11.48	7
Other	3.28	2

### Flavors and Attractive Features of Disposable ENDS

**Fig. 2:**
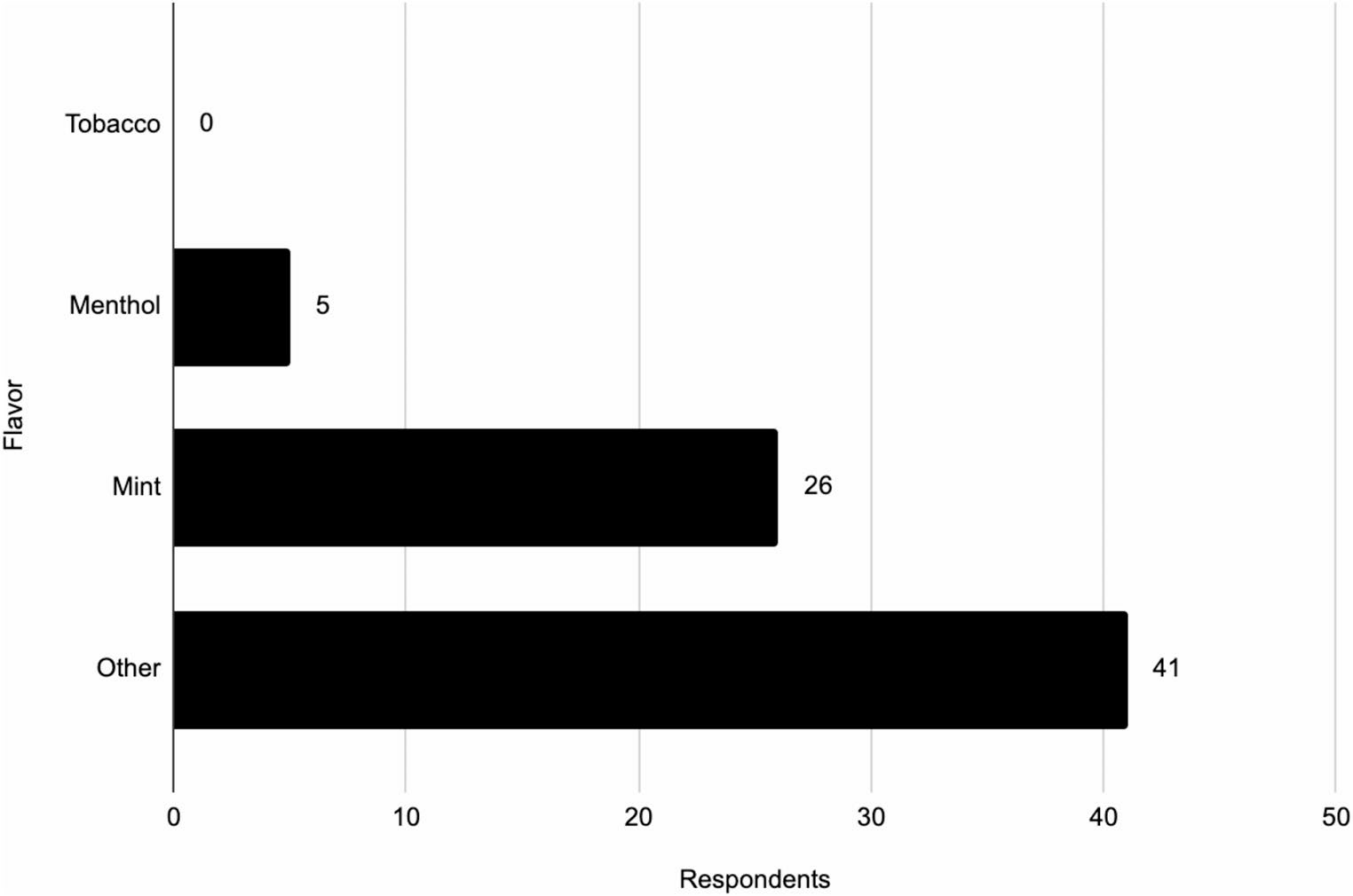
Flavor of disposable ENDS used by underage respondents (*N* = 72)

Figure 2 shows the responses from underage participants on the flavor of disposable ENDS they most commonly use. This question revealed that no respondents use tobacco flavored disposable ENDS and only 6.94% (5/72) use menthol flavors. This means that 93.06% (67/72) of underage disposable ENDS using respondents use flavored disposable ENDS, according to the FDA’s definition of a flavored ENDS product which is any flavor other than menthol or tobacco. Furthermore, 56.94% (41/72) of these respondents indicated they typically use a flavor that is not tobacco, menthol, or mint which likely corresponds to a fruity or candy-like flavor that are very commonly found among disposable ENDS.

Table 4 shows the results of the survey question that asked participants to select the single most attractive aspect of disposable ENDS that influences them to choose disposable ENDS over other ENDS devices. “Convenience/ease of use” stands out among these results as the most attractive reported aspect of disposable ENDS with 45.76% (27/59) of responses. “Flavor” and “Your friends use them” followed as the second and third most attractive factors with 20.34% (12/59) and 22.03% (13/59) of responses respectively. 0.00% (0/59) of respondents selected “Helps you quit cigarettes” as the most attractive aspect of disposable ENDS indicating that underage respondents are not using disposable ENDS to quit cigarettes. Furthermore, cost, variety, and ease of procurement were not commonly reported to be the most attractive aspect of disposable ENDS.

**Table 4:** The most attractive aspect of disposable ENDS according to underage respondents that identified disposable ENDS as usual device (*N* = 59)

Attractive Aspect	Percent of Responses	Number of Responses
Convenience of Use	45.76	27
Your Friends Use Them	22.03	13
Flavor	20.33	12
Ease of Procurement	5.08	3
Wide Variety of Products	3.39	2
Cost	1.69	1
Other	1.69	1
Helps You Quit Cigarettes	0.00	0

### Disposable ENDS Brand Popularity

**Fig. 3:**
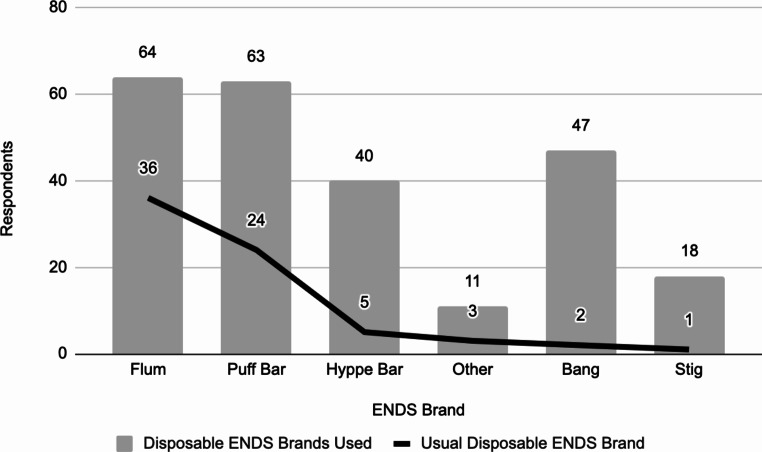
All disposable ENDS brands used (*N* = 72) and usual disposable ENDS brand of underage respondents (*N* = 71)

Figure 3 shows the distribution of all the brands of disposable ENDS that underage users indicated they had used. Underage respondents’ most commonly used disposable ENDS brand as indicated on the survey is also shown in Fig. 3.

### Underage Acquisition of Disposable ENDS

**Fig. 4:**
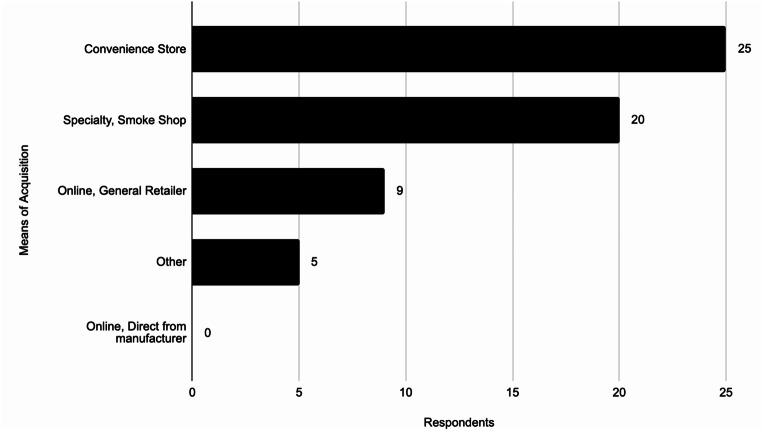
How underage users acquire disposable ENDS (*N* = 59)

When underage respondents who indicated disposable ENDS as their usual device were asked where they most commonly acquire their disposable ENDS, the majority indicated they usually acquire ENDS at a convenience store or a specialty smoke shop (Fig. 4).

## Discussion

Current literature on ENDS use has an emphasis on persons under the age of 18, with no specific emphasis on disposable ENDS. While ENDS use by persons under 18 years old is significant and concerning, it is also important to note that ENDS use by anyone under the age of 21 is illegal in the U.S. Data on underage ENDS use, specifically disposable ENDS, is also needed because it is evident that these devices are popular among persons under 21. The data collected in this survey is valuable in revealing disposable ENDS use by persons aged 18–20 and may give insight on users under 18 since they exhibit similar behavioral patterns in how they use and acquire ENDS.

While using ENDS a minimal number of times is not likely to result in any significant health complications, the frequency and willingness of underage persons to try ENDS is putting a portion of this population at risk of becoming dependent on nicotine and possibly regular ENDS users [6]. It is hypothesized that many respondents who tried ENDS while underage were introduced to the devices by peers and motivated to use ENDS either by a misplaced perception of safety, peer pressure, or a desire to be accepted.

While this survey did not have participants under 18, self-reported data on age of initiation to ENDS revealed important information that is not found in current literature. While the NYTS gathers patient demographic data including age, there is no specific question to determine the age participants first used ENDS. This survey determined that 99.19% (123/124) of respondents used ENDS for the first time while under the age of 21. The results specifically show that 75.27% (70/93) of adolescents began using ENDS between the ages of 15–18, and 5.38% (5/93) as early as 13–14. Identifying the at-risk ages for initiation to ENDS products is important for developing educational resources tailored for each group. Regardless, this data is concerning in showing young ages of initiation to ENDS that, for many participants, carries into early adulthood and creates long-term nicotine addiction.

Furthermore, 71.57% (73/102) of underage respondents who had used ENDS indicated that they had used disposable ENDS at least once. This result showed the extent to which disposable ENDS have penetrated the underage ENDS-using culture, despite most lacking FDA pre-market authorization. The data revealed that 49.02% (50/102) of underage ENDS users first used pod-based ENDS, but the most popular ENDS devices were disposables. This confirms trends seen after 2019 when the FDA enacted restrictive regulations on pod-based ENDS, such as JUUL, and disposable ENDS overtook pod-based ENDS as the most popular device among young people. Based on this information, it is hypothesized that many survey respondents first used ENDS around 2019 when JUUL was popular but switched to disposable ENDS after JUUL was forced to make changes to their products including limiting their flavors. However, 33.33% (34/102) of underage respondents first used disposable ENDS, likely because many started vaping after JUUL’s popularity decreased and disposable ENDS’ popularity began. Nonetheless, with disposable ENDS being the most popular device among underage ENDS users and contributing a large, and likely growing, proportion of first ENDS device used, it is evident that disposable ENDS are the key device contributing to underage ENDS use and subsequent nicotine addiction.

This hypothesis is supported by the data which shows the age participants first used disposable ENDS. The most common age group in which participants first used disposable ENDS was 17–18, 48.39% (45/93), but the most common age group participants first used any ENDS product was 15–16, 43.55% (54/124). Evidence from the literature reviews lends to the idea that this is due to the novelty of disposable ENDS as a popular device type rather than youth not being introduced to disposable ENDS at young ages. This hypothesis is further supported by the data wherein many respondents did not use disposable ENDS until after 2017. Since survey respondents were all over the age of 18, many of them were likely over age 16 when disposable ENDS gained popularity. Thus, it is hypothesized that disposable ENDS will become a more common first device and used in the earlier teenage years if measures are not taken to significantly reduce underage consumption of disposable ENDS.

Compared to the NYTS, which only collects data on frequency of use over the past 30 days in number of days used, this survey collected data on frequency of disposable ENDS use throughout a single day, which is more specific and representative of ENDS use [4]. Alarmingly, 44.26% (27/61) of respondents indicated they used ENDS 10 or more times a day, causing more nicotine uptake into their system which may lead to greater or quicker symptoms of nicotine dependence. Interestingly, around 11.48% (7/61) of disposable ENDS users indicated that they only used ENDS socially implying that ENDS are commonly present in social situations for underage people. Unlike the NYTS, this survey distinguished between social and regular ENDS users, data for which may be used or built upon to properly educate young people that addiction and other negative health consequences can still result from occasional use.

Much evidence has been collected on flavored ENDS use among youth and how flavors increase the risk for youth use of tobacco products by reducing perceptions of harm [7]. However, no data specific to flavored disposable ENDS has been collected and they continue to be overlooked by regulators. The survey revealed that 93.06% (67/72) of underage disposable ENDS users use flavored disposable ENDS, any flavor besides menthol or tobacco. No further distinction of the answer choice “any other flavor” was made because a review of ENDS websites revealed hundreds of flavors.

While flavors have been banned for pod-based ENDS, flavored disposable ENDS may be a key reason why underage users have switched from pod-based to disposable ENDS. This raises regulatory questions as to why flavored pod-based ENDS are banned, but sale of flavored disposable ENDS is not explicitly prohibited. Nevertheless, it is important to acknowledge that most disposable ENDS companies have not submitted PMTAs, and the FDA would possibly refuse to grant premarket authorization to many disposable ENDS because their flavors are appealing to underage users. Some states have already taken regulatory matters into their own hands. For instance, California passed Proposition 31 in November 2022 which officially prohibited the sale of almost all flavored tobacco products including ENDS [8].

While flavorings have been shown to be a risk factor for youth ENDS use, this study sought to identify other factors that make disposable ENDS attractive to underage users. Underage disposable ENDS users identified many attractive features including convenience/ease of use, flavor, cost, wide variety of products, and ease of procurement. Underage respondents selected many of the same answer choices for the most attractive features of disposable ENDS, but convenience/ease of use was a clear leader as the most attractive aspect. This result supports the hypothesis that the ease of using disposable ENDS is a driving factor contributing to their popularity since they only require a single device allowing participants to begin vaping after a single purchase.

The second most attractive aspect of disposable ENDS for underage respondents was that their friends use them, which further shows the social pressures on underage persons to use disposable ENDS. Whether this indicates that direct social influences, such as peer pressure; indirect social influences, such as a desire to be accepted; or other social cues are the reason respondents selected this choice would require further, more specific research on this topic, but the results are indicative of a link between social influences and disposable ENDS use regardless.

The third most attractive aspect of disposable ENDS was flavor, which reinforces the literature. It is hypothesized that flavors are specifically identified as an attractive feature of disposable ENDS because they can no longer be obtained for pod-based ENDS. Regulatory reform against pod-based ENDS, to reduce flavors and attractive features for adolescents, has been quite successful. This could be due to the concentration of popularity of pod-based ENDS on just a few brands whereas the popularity of disposable ENDS is spread across many brands, making it difficult or ineffective to bring regulatory action against the industry. However, the FDA could have more grounds for punishment of disposable ENDS if they officially banned flavors for all ENDS products.

Only a select number of brands were provided as answer choices on this survey to avoid overwhelming respondents or causing noncompliance. Even so, this survey listed more disposable ENDS brands than the 2023 NYTS. Thus, this survey revealed unknown information about the competitive landscape of the disposable ENDS industry and its popularity among underage users. While much of the FDA’s focus on the disposable ENDS industry has targeted Puff Bar, there appears to be a need for similar action against other companies. The most common disposable ENDS brands used by respondents were Puff Bar, Hyppe Bar, Flum, and Bang with others writing in brands not listed.

While many underage respondents had used Puff Bar, the results of the survey revealed that Flum was the most popular usual disposable ENDS brand. It is hypothesized that Puff Bar was very popular when this survey was first deployed but declined in popularity as they were more strictly regulated and forced to remove nicotine from their products. Thus, many other brands of disposable ENDS have remained popular because they have not received any action from the FDA. However, some sources suggest that the popularity of disposable ENDS brands may vary greatly by region of the U.S., so this study’s brand popularity data may be specific to Southern California. Nonetheless, these results do support the popularity of many disposable ENDS brands and a possible decline in the popularity of Puff Bar after diligent regulatory enforcement led them to remove nicotine from their products.

The survey found 76.27% (45/59) of underage respondents acquired disposable ENDS from in-person stores such as convenience stores or smoke shops. While these underage persons should not technically be able to buy ENDS from these stores, it is clear that underage people are purchasing disposable ENDS. This survey did not have a method for determining how underage persons are able to purchase disposable ENDS from these stores, but it is presumed that these stores either do not check purchasers’ IDs or underage persons use fake IDs to obtain ENDS. 15.25% (9/59) responded that they obtained the devices online from general retailers, which should also be verifying age of purchasers. This emphasizes the need for regulatory bodies and law enforcement to ensure that retailers, both online and in-person, are properly checking the identification of all ENDS purchasers to reduce underage acquisition of tobacco products.

The results of this study were significant in revealing important information about underage disposable ENDS use, much of which is not present in any current literature. These results may be used by regulatory bodies, such as the FDA, to make meaningful regulatory reform to reduce the attractive aspects of disposable ENDS that promote underage use and by educational institutions to effectively educate young people against disposable ENDS use. However, it is important to acknowledge that youth, under 18, disposable ENDS use is not the same as underage, under 21, use. While it is believed that the results of this study could be reasonably generalized to youth disposable ENDS use, this assumption is speculatory. Thus, it cannot be said that the results would be the same for youth disposable ENDS users who may have slightly different perceptions and patterns of use. However, it is believed that regulatory and educational reform based on these results would likely be effective in reducing both underage and youth disposable ENDS.

Furthermore, it is important to recognize that all respondents used for data analysis in this study were college students, so a large portion of the underage population, non-college students, was not included. Thus, these results are biased toward underage college students’ perceptions and patterns of disposable ENDS use, and the same results may not be found in a more general sample of persons 18–20. Nevertheless, these results are believed to have relatively uniform results for the age group 18–20. Last, many survey respondents were likely California residents since the majority of survey deployment occurred at California universities, most specifically the University of Southern California, so different results may be found if this survey was conducted in other parts of the country. However, due to our diverse sample, it is believed that many of the results regarding disposable ENDS patterns of use may be cautiously generalized to underage persons in other areas of the U.S. The biggest difference would likely be regarding brand popularity as research suggests many regions have different disposable ENDS available in stores. However, to accurately assess the generalizability of our results, future research should aim to replicate these findings using a nationally representative sample. This would help to confirm whether the patterns observed in our study are consistent across different regions and demographic groups in the U.S. young adult population.

As mentioned above, the most significant limitation of this study is that the convenience sample consisted only of college students, most of whom attended the University of Southern California. Ideally, a more random and locationally diverse sample would have been used, so future studies incorporating a larger, nationwide cohort would be beneficial to confirm these results and draw more generalizable conclusions. The survey design also limited the study in that it relied on self-reporting and utilized a minimal selection of questions to deduce as much information as possible while reducing, but not eliminating, non-response bias from college students who may have little motivation to complete the survey. Furthermore, the decision was made to utilize descriptive statistics, rather than detailed statistical analysis, when describing survey results to emphasize their raw significance in revealing notable patterns in college students’ patterns of use with ENDS.

## Conclusion

Nicotine addiction among young people remains a major public health issue in the U.S., and disposable ENDS are the main device furthering this epidemic. The results of this survey confirm the popularity of disposable ENDS among the underage responders. Further, our results provide additional support to the hypothesis that disposable ENDS have gained popularity after a decline in popularity of pod-based ENDS, such as JUUL, following FDA regulations banning flavors in such devices. While no studies have yet been able to confirm the possible long-term health consequences of regular ENDS use, many ENDS have known toxins, carcinogens, and highly addictive nicotine, which coupled with high frequency of disposable ENDS use is furthering long-term nicotine addiction.

According to this study, FDA’s efforts to regulate JUUL have decreased pod-based ENDS use among underage users from our student sample, but disposable ENDS remain popular. Numerous disposable ENDS brands are operating without FDA premarket authorization, and the results from our survey confirm the popularity of additional brands, such as Flum, among underage respondents. Nonetheless, the FDA has proven that through relentless regulatory action, such as that brought against Puff Bar, the disposable ENDS industry can be properly regulated. The FDA must expand their regulatory efforts to hold all disposable ENDS brands and tobacco products to the same standards and hold them accountable for their contribution to underage nicotine addiction [7].

Flavors and ease of use were two of the many aspects that attracted the young people in our survey to disposable ENDS and helps to substantiate their unfitness for premarket authorization. Our data adds to the burgeoning data available and should aid the FDA to make a case for the removal of all disposable ENDS from the market unless proper premarket authorization is provided. Furthermore, social influences appear to be a significant factor leading to underage disposable ENDS use, so improved education to young people on the negative aspects of ENDS use and nicotine addiction are necessary to reduce underage uptake and subsequent social pressures to use the devices. Additionally, since underage people acquire disposable ENDS most commonly from in-person locations, FDA should collaborate with local law enforcement to halt the sale of unauthorized ENDS products from convenience stores and vape shops as well as online ENDS retailers.

## Data Availability

Data is provided within the manuscript.
